# The Decentralisation of Great Cities

**Published:** 1898-11-26

**Authors:** 


### THE DECENTRALISATION OF GREAT CITIES.

Although considerable newspaper prominence has been given of late to the difficulty experienced by working men with large families in obtaining house room in certain districts south of the Thames, the trouble is not one of to-day, nor of one locality alone. It has been growing for years past, and it exists in a marked degree all over the central portions of the metropolis. Nor does it affect those only who live by manual labour. Everyone whose work has to be done within the circle finds himself on the horns of a dilemma; either he must spend an exorbitant proportion of his earnings in mere rent, or he must spend an exhausting proportion of his daily toil in trailing his weary body to and fro between his place of work and his so-called home, or, to speak with more accuracy, his sleeping place, for that is about what a worker's home becomes when it is far removed from his work. And with new "improvements," and a growing strictness in the application of sanitary rules by the authorities, especially such rules as have reference to overcrowding, the difficulty increases daily, and must continue to do so. It becomes then of the greatest importance that in seeking remedies for this state of affairs we should go upon sound principles, and especially that we should not employ methods which, while doing good in one direction, may do an equal amount of evil in another. The real question is whether to provide increased house accommodation in the centre, or more work in the suburbs; whether to centralise or to decentralise. Many are already to be found who will declare that the difficulty is due to intermeddling sanitation, while nothing is easier than to say that the want of houses can only be remedied by the simple plan of building more. If, however, one goes to the root of the matter, one sees that in the causation of the overcrowding of towns certain economic laws are in operation by which what may be called a vicious circle is produced, and that if any remedy is to be found it must be something which will tend to break up this "vicious circle" rather than intensify it, as mere building would tend to do. People crowd in towards the centre because the work is there; manufactures crowd in toward the centre because labour is there. The more work there is to choose from the better is labour satisfied, while the more labour there is to pick out of the better is capital satisfied; so both press inwards, and, unless the authorities make a firm stand about sanitary matters and fix a rigid limit to overcrowding, the struggle for existence between man and man must beat down the standard of living to the lowest level which is compatible with mere existence. Nor is the difficulty relieved by the municipality building more houses. For a moment the pressure may seem to be lessened, but in fact the dwellings so provided, if let for less than the market price, are but a gift to capital of so much labour at a cheaper wage than it otherwise would have to pay, and thus, by drawing work towards a district where such subsidised labour can be obtained, tend to increase the very evil they were meant to cure. Thus between the worker and his employer there is a mutual action and reaction which must always make it impossible to relieve the congestion by mere building.

What is wanted is to get the people into the suburbs and, if possible, to draw the manufacturing industries from the overcrowded centre. If we thought that it was a divine law, and immutable, that people in Southwark, for example, should be packed together more than two hundred on each acre, we would say let us build and at least make the poor wretches as comfortable as possible. But such is not the case, and we believe that there is a better future for the worker than to live in a block dwelling. To us the problem appeals as a question of health, and what we would like to see is a policy of spread rather than a policy of concentration—a policy of cottages and gardens rather than one of blocks and staircases. Far better that municipalities should make use of the streets, which are their own, for the full development of every possible means of communication with the suburbs, than spend the money of the ratepayers on the erection of workmen's dwellings in central localities. Block dwellings built in the centre cause overcrowding on the land, not merely by the land they occupy, but by encouraging the concentration of all means of earning a living; while every working-class colony firmly established in the suburbs tends to draw to itself industries which will give occupation to its members near at hand. The aim should be to break up that centralisation of trade and industry which, by causing concentration of population—crowding of houses on land and huddling of people in houses—is the cause of so much disease and death in the central parts of London.

